# Association between *CRP* and *TNF-α* genes Variants and Cardiovascular Heart Disease in a Mexican Population: Protocol for a Case-Control Study

**DOI:** 10.3390/ijerph13010103

**Published:** 2016-01-06

**Authors:** Yazmín Hernández-Díaz, Carlos Alfonso Tovilla-Zárate, Isela Juárez-Rojop, María Lilia López-Narváez, José Francisco Álvarez-Cámara, Thelma Beatriz González-Castro

**Affiliations:** 1 División Académica Multidisciplinaria de Jalpa de Méndez, Universidad Juárez Autónoma de Tabasco, Jalpa de Méndez, Tabasco 86205, México; yazmin.hdez.diaz@gmail.com; 2 División Multidisciplinaria de Comalcalco, Universidad Juárez Autónoma de Tabasco, Comalcalco, Tabasco 86650, México; alfonso_tovillaz@yahoo.com.mx; 3 División Académica de Ciencias de la Salud, Universidad Juárez Autónoma de Tabasco Villahermosa, Tabasco 86150, México; iselajuarazrojop@hotmail.com (I.J.-R.); paco_cca@hotmail.com (J.F.A.-C.); 4 Hospital General de Yajalón. Secretaría de Salud, Yajalón, Chiapas 29932, México; dralilialonar@yahoo.com.mx

**Keywords:** CAD, CRP, TNF-α, association studies, Mexican population

## Abstract

Background: The C-reactive protein (*CRP*) and the tumor necrosis factor-alpha (*TNF-α*) are considered markers of inflammation and have been shown to predict the risk of incident cardiovascular events. However, few studies have undertaken a comprehensive examination of SNPs (single nucleotide polymorphisms) of the *CRP* and *TNF-α* genes; due to this, we will present a protocol study to evaluate the role of the *CRP* and *TNF-α* genes in Mexican individuals. Methods/design: we will perform a case-control study to explore the *CRP* and *TNF-α* genotype distribution as well as the serum influence of rs1800947, rs1130864, rs2794521 and rs1205 (polymorphisms of the *CRP* gene) and rs361525, rs1800629, rs1799724, rs1800630, rs1799964 (of the *TNF-α* gene) in Mexican individuals who present coronary artery disease. Ethics and dissemination: a written informed consent will be obtained from all the participating subjects. An article detailing the results of the study will be submitted for publication in an international peer-reviewed journal, in accordance with STROBE criteria.

## 1. Introduction

Coronary artery disease (CAD) has often been considered a problem of wealthy, industrialized nations [1,2]. In fact, as the leading cause of death worldwide, CAD has now a major impact not only in developed nations but also in low and middle-income countries [3,4,5,6]. As the time passes, the interest for genetic research on common CAD has progressively moved to the search of possible genes which may play an important role in the development or detection of the disease [7]. In the pathogenesis of CAD, inflammation plays a crucial role through its contribution to atheroma formation and plaque rupture [8]. However, despite enormous advances in the understanding of the genetic basis; currently, conclusive markers have not yet been established [9,10]. Among the various gene association studies of CAD the most reported genes are the C-reactive protein (*CRP*) and the tumor necrosis factor-α (*TNF-α*) [11,12,13]. These genes encode inflammatory markers and have variants that regulate their expression and are potential risk factors for CAD. There is strong evidence that CRP is a powerful predictor of incident cardiovascular events [14,15]. The human *CRP* gene lies on chromosome 1q21–1q23, spans approximately 1.9KB and contains two exons, which are joined by a 280 base pair intron [16,17]. Studies have reported that polymorphisms within the *CRP* gene are associated with plasma CRP levels [18,19]. The most studied polymorphisms of the *CRP* gene in association with CAD are the 1059G>C (rs1800947), 1444C>T (rs1130864), 717A>G (rs2794521) and 3872G>A (rs1205) variants [19,20]. It has been observed that these four principal variants increase or decrease the CRP serum levels and are rapidly up-regulated by inflammatory cytokines. Moreover, CRP levels are a sensitive indicator of inflammation and a marker of CAD [21,22]. Tumor necrosis factor-alpha (*TNF-α*) also plays a key role in cardiovascular pathophysiology. *TNF-α* gene is located on chromosome 6 (p21.1–p21.3) and consists of 2.76 kb distributed across its promoter region, four exons, three introns and untranslated regions 5′ and 3′ [23,24]. Genetic variants in the *TNF-α* promoter region are reported to be associated with the TNF-α serum levels, these levels are correlated with the first-time cardiovascular disease and are also a marker for recurrent coronary events after a previous myocardial infarction [25,26]. Numerous polymorphisms of the *TNF-α* gene have been reported, including 308G>A (rs1800629), 238G>A (rs3615525), 857C>T (rs1799724), 863C>A (rs1800630) and 1031T>C (rs1799964) [27,28]. The polymorphisms 308G>A (rs1800629) and 238G>A (rs3615525) are the most studied variants of *TNF-α*, because several studies have found that patients with the A allele show high levels of TNF-α in serum, modifying the risk of developing CAD [29,30]. However, the association found between CAD and these genes must be validated using larger and independent sample populations, using case-case control studies, in order to prevent spurious associations [31,32] and better understand the incidence of cardiovascular events, a case-control study is contemplated.

## 2. Protocol Objectives

The present study is designed as a protocol to determine the association between CAD and the polymorphisms of the *CRP* and *TNF-α* genes: rs1800947, rs1130864, rs2794521, rs1205 and rs361525, rs1800629, rs1799724, rs1800630, rs1799964, respectively. It will also investigate whether any polymorphisms of the *CRP* and *TNF-α* genes affects the serum levels of CRP and TNF-α proteins in Mexicans patients with CAD confirmed by angiography.

## 3. Methods

### 3.1. Study Patients

**Figure 1:**
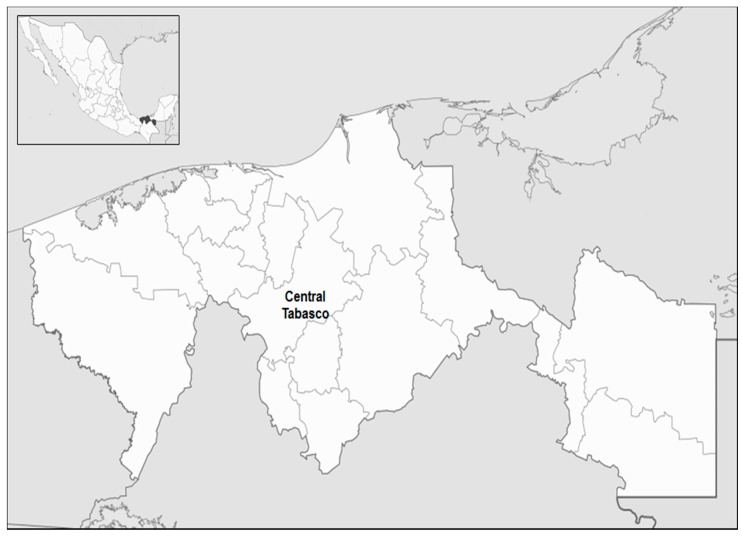
Tabasco state map; location of the population to be studied.

The sample will be recruited from the Cardiology Service of the Mexican Social Security Institute (IMSS, initials in Spanish), clinic UMF 46, Tabasco, Mexico (Figure 1). The study group will consist of 500 patients with CAD, 500 controls with patent coronaries and 500 healthy subjects without any familiar history of CHD (coronary heart disease). All participants have to be Mexican, with at least two ascending generations born in Mexico, mainly residing in Central Tabasco. Any person presenting rheumatologic disorders, malignancy, infection or overt heart failure will be excluded from the study.

We will collect the following information from the participants: age, sex, BMI (body mass index) and risk factors for atherosclerosis (diabetes, hypertension, smoking, *etc.*). Blood analyses will include: CRP and TNF-α serum levels, complete blood count, blood sugar, biochemistry and lipid profiles; patients without complete demographic data and/or inappropriate processing laboratory specimens will be excluded from the study. 

### 3.2. Phenotype Definition in Study Patients

CAD will be defined as the presence of stenosis of more than 50% of the luminal diameter in at least one significant coronary artery determined by coronary angiography; the subjects who show patent coronaries will be enrolled as controls. The CAD patients group will go under a second analysis in order to classify them in two groups: (a) multi-vessel disease group (two or more coronary arteries affected) and (b) single-vessel disease group (one coronary artery affected). Moreover, to reveal the genetic effect that CAD may have on acute myocardial infarction (AMI), *enunciado inconmprensible, recomeindo:* to observe a possible genetic influence of the *CRP* and *TNF-α* polymorphisms over acute myocardial infarction (AMI), we will obtain information on CAD patients including: new onset AMI (according of the European Society of Cardiology −ESC− and American College of Cardiology −ACC− [33]) and historical AMI (medical record during hospitalization). Patients having chronic stable angina will be classified as controls. 

### 3.3. Ethics Statements and Dissemination

All subjects included in the study will sign an informed consent after explaining in detail the objectives of the study to the patients and their relatives; the participants will not receive any economical remuneration. This study has already been approved by two Ethics Committees: one from the Mexican Institute of Social Security, clinic UMF.49 (IMSS2013121) and the Research Committee of the University of Tabasco, México (DAMJM-UJAT; P.O.A. 20110237). Also, the study will be performed in accordance with ethical standards convened in the 1964 Declaration of Helsinki. An article detailing the results of the study will be submitted for publication in an international peer-reviewed journal, in accordance with Strengthening the Reporting of Observational Studies in Epidemiology (STROBE) criteria [34].

### 3.4. Genotype Assays

A peripheral blood sample will be collected from each participant (CAD and patent coronaries). Also, genomic DNA from leukocyte blood sample will be extracted using a modified version of the protocol by Lahiri *et al.* [35]. The polymorphisms of the *CRP* and *TNF-α* genes chosen for the study will be: rs1800947, rs1130864, rs2794521 and rs1205, as well as rs361525, rs1800629, rs1799724, rs1800630, rs1799964, respectively (Table 1). These variants will be analyzed using the polymerase chain reaction (PCR) end-point method. The final volume of the PCR reaction will be 5μL and will consist of 20 ng genomic DNA, 2.5 Fluorescence Labeling (FL) TaqMan Master Mix and 2.5 FL 20 × Assay. Next, the amplification will be performed in 96 well plates using the TaqMan Universal Thermal Cycling Protocol. Fluorescence intensity will be measured in a 7500 Real-Time PCR system using SDS 2.1 software (Applied Biosystems, Foster, CA, USA). To confirm the consistency of the results, all genotyping will be carried out blind to patient outcome and random blind duplicates will be run for the 30 percent of the analyses.

### 3.5. Statistical Analysis 

First, we will evaluate the Hardy-Weinberg Equilibrium (HWE) using Pearson’s goodness-of-fit chi-squared test. We will use measures of central tendency with the mean ± SD and median with minimum and maximum values in accordance with their distribution. Moreover, continuous variables will be compared using Student’s *t* test or Mann-Whitney test. Also, the chi-squared test or Fisher’s exact test will be used to compare genotype and allele frequencies between controls and cases, using the following models: co-dominant (major allele homozygotes *vs.* heterozygotes and major allele homozygotes *vs.* Minor allele homozygotes), dominant (major allele homozygotes *vs.* heterozygotes + minor allele homozygotes), recessive (major allele homozygotes + heterozygotes *vs.* minor allele homozygotes), additive (major allele homozygotes *vs.* heterozygotes *vs.* minor allele homozygotes) and heterozygous advantages (homozygote for the minor allele + homozygote for the major allele *vs.* heterozygote). For all the association analyses the level of significance will be set at *p* < 0.05, however the *p* value will be corrected according with the number of specificities tested and the comparisons made; the confidence interval will be set at 95%. Logistic regression analysis will be performed to estimate the risk of CHD between cases and controls against the CRP and TNF-α gene variants. Statistical calculations will be performed using the SPSS software, *version* 15. The Haploview 4.1 software will be used to construct haplotypes and linkage disequilibrium (LD) between polymorphisms. Finally, the sample size will measure of association using Quanto version 1.2 software (http://biostats.usc.edu/Quanto.html). The power of the analysis will 0.99 (minor allele frequency = 0.34; type od model: log-additive; genetic relative risk = 1.5).

**Table 1:** Single nucleotide polymorphisms (SNPs) of the C-reactive protein (CRP) and tumor necrosis factor-alpha (TNF-α) genes in this protocol study.

SNP ID	Alleles	Region	Localization
*CRP* gene			
rs1800947	G/C	Coding exón	+1059
rs1130864	C/T	3′ UTR	+1444
rs2794521	A/G	Promoter	−717
rs1205	G/A	3′ UTR	+3872
*TNF-α* gene			
rs361525	G/A	Promoter	−238
rs1800629	G/A	Promoter	−308
rs1799724	C/T	Promoter	−857
rs1800630	C/A	Promoter	−863
rs1799964	T/C	Promoter	−1031

## 4. Discussion

It is well known that the first causes of mortality in adults worldwide are cardiovascular diseases; particularly, coronary artery disease [36]. For Mexicans, most of the information on coronary risk factors comes from European or American studies, some of them made over 50 years ago [37,38]. The identification of coronary risk factors is not a simple task; biological markers do not completely explain the CAD complex pathology [15]. The recent and rapid development of molecular genetics in CAD has created a new understanding of this pathogenesis. In this way, a growing number of studies report that inflammation plays a crucial role in this pathogenesis; it has been reported that during the acute phase, the reactant C-reactive protein can be predictive of future cardiovascular events, including myocardial infarction (MI), ischemic cardiac events or sudden death among patients with angina pectoris [39]. As a marker of systematic inflammation, it is not yet known whether elevated CRP levels are linked either to the inflammatory response or to genotype distribution of *CRP* gene variants [37]. Other proinflammatory cytokine is the tumor necrosis factor-alpha, several evidence show that is a key contributor in the development, progression, and complications of atherosclerosis [40]. Moreover, high concentrations of plasma-soluble TNF-α are considered a key feature in cardiovascular diseases, such as atherosclerosis, CAD and MI [29,41], therefore it is also a marker for recurrent coronary events [42]. Due to this evidence we propose to analyze the distribution of the most studied variants of *CRP* gene (rs1800947, rs1130864, rs2794521 and rs1205) and *TNF-α* gene (rs361525, rs1800629, rs1799724, rs1800630, rs1799964) in a Mexican population in association with CAD. On the other hand, we want to observe the role of the genotype distribution in CRP and TNF-α serum levels, which are considered a marker of CAD. The outcomes of the study will provide new and unique information of the *CRP* and *TNF-α* genes participation in CAD, in Mexican individuals. This type of study is relevant for the Mexican population because the CAD has increased considerably (similar to European populations) and it cannot be explained merely by environmental factors (such as smoking and lack of physical activity among others), furthermore, the investigation of probable genetic markers of CAD in Mexico has not advance as much as in European countries [43,44,45]. Although there are several reports about CAD, to date there are no studies to evaluate the genetic role of *CRP* and *TNF-α* in CAD. The purpose of the genetic research in the Mexican population will try to establish possible markers in the pathogenesis of CAD and the results of this study will be of great scientific relevance. 

The limitations of this study will be the lack of genetic ancestry evaluation of the participants, as well as the lack of search for a possible effect that could cause the stratification population. In conclusion this protocol study will try to unravel the role of some *CRP* and *TNF-α* genes variants; it will also analyze how such polymorphisms are involved in CRP and TNF-α serum levels in subjects with CAD. Therefore, the outcomes of this protocol will provide information to gain a better insight into CAD, which is considered a leading cause of death in Mexico and the world.

## 5. Conclusions

The cardiovascular heart disease has increased in Mexico, and its pathology can not be only explained by environmental effects. The role of genetic risk factors has been considered in several studies and this is an important point that needs to be considered by all health professionals. Due to this we considered that the development of this protocol can provide valuable data to the goal of a prevention through genetics markers. 
